# Adiponectin, leptin and IL-1 β in elderly diabetic patients with mild cognitive impairment

**DOI:** 10.1007/s11011-015-9739-0

**Published:** 2015-10-02

**Authors:** Malgorzata Gorska-Ciebiada, Malgorzata Saryusz-Wolska, Anna Borkowska, Maciej Ciebiada, Jerzy Loba

**Affiliations:** Department of Internal Medicine and Diabetology, Medical University of Lodz, ul. Pomorska 251, 92-213 Lodz, Poland; Department of General and Oncological Pneumology, Medical University of Lodz, ul. Kopcinskiego 22, 90-153 Lodz, Poland

**Keywords:** Adiponectin, Diabetes, IL-1 β, Leptin, Mild cognitive impairment

## Abstract

The aim of the study was to determine the serum levels of adiponectin, leptin and IL-1 β in elderly diabetic patients with and without mild cognitive impairment (MCI) and to examine the associations of these markers with clinical and cognitive parameters. A biochemical evaluation was performed of 62 seniors with type 2 diabetes (T2DM) and MCI, and 132 seniors with T2DM but without MCI (controls). Serum leptin and IL-1 β levels were higher and adiponectin concentration was lower in MCI patients than controls. In MCI subjects, adiponectin level was negatively correlated with leptin, IL-1 β levels and BMI. Leptin concentration was correlated with IL-1 β level. Univariate logistic regression models revealed that the factors which increased the likelihood of diagnosis of MCI in elderly patients with T2DM were higher levels of HbA1c, leptin, IL-1 β and triglycerides, as well as lower levels of adiponectin and HDL cholesterol. Similarly, previous CVD, hypertension, hyperlipidemia, retinopathy, nephropathy, hypoglycemia, longer duration of diabetes, increased number of co-morbidities, older age, fewer years of formal education were found to be associated with MCI. The multivariable model indicated fewer years of formal education, previous CVD, hypertension, increased number of co-morbidities, higher HbA1c and IL-1 β levels and lower adiponectin level. Elderly diabetic patients with MCI have higher levels of leptin and IL-1 β and lower levels of adiponectin. Further prospective studies are needed to determine the role of these markers in the progression to dementia.

## Introduction

Type 2 diabetes (T2DM) is a chronic disease with well-established cross-sectional and longitudinal associations with cognitive impairment (Cukierman et al. 2005; Munshi et al. 2006; Ott et al. 1996). Mild cognitive impairment (MCI) is defined as a transition stage between normal aging and dementia. Many studies indicate that T2DM is a risk factor for MCI (Gorska-Ciebiada et al. 2014; Cheng et al. 2012; Kodl and Seaquist 2008). On the other hand aging itself is also related to cognitive dysfunction: the prevalence of MCI in subjects aged 70 years and older is 14–18 % (Petersen et al. 2009; Sinclair et al. 2000).

Recent studies have suggested that inflammation may play an important role in the presence and development of MCI (Hermida et al. 2012; Gorska-Ciebiada et al. 2015; Umegaki et al. 2011; Barnes et al. 2006).

IL-1 β is one of the proinflammatory cytokines involved in damage of islet beta cells (Palmer et al. 1989). One study performed in rats found that pancreatic tissues expressed elevated levels of IL-1β, and IL-1β-driven inflammatory cascade in diabetes (Ehses et al. 2009). This imbalance between IL-1β and IL-1 receptor antagonist leads to pancreatic islet inflammation and release of insulin. Using a recombinant human IL-1 receptor antagonist is one of the novel anti-inflammatory treatments which lead to the correction of beta cells dysfunction and decreased systemic inflammation in T2DM (Akash et al. 2012). There is also strong evidence that inflammation can pay a crucial role in the brain during Alzheimer’s disease. Lue et al. (2012) propose that diabetes causes macro and microvascular complications which impair blood–brain barrier function and endothelial cell activation. Microglia in the brain became activated by vascular injury and start a process of functional and morphological transformation and degeneration (Lue et al. 2012). Although many studies show increased expression of inflammatory mediators and microglial activation in MCI and dementia it has been hypothesized that pro-inflammatory cytokines are derived from peripheral sources like adipose tissue. It is possible that central and peripheral inflammation pathways are responsible both for the neurodegeneration process. The adipose tissue produces many pro-inflammatory cytokines including IL-1β, IL 6, IL 18 and TNF-α (Monteiro and Azevedo 2010). The levels of these mediators were elevated in peripheral blood of patients with Alzheimer Disease (Swardfager et al. 2010). Adipose tissue also produces also an pro-inflammatory hormone: leptin. T2DM and obesity is associated with hyperleptinemia and subsequent leptin resistance. Studies suggest that leptin can act as a neuro-inflammatory signal, one which may attract different immune cells and stimulate the production of such cytokines as IL-1β (Lam and Lu 2007). The leptin receptors are present in microglia and regulate the synthesis of IL-1β. High levels of plasma leptin are associated with the production of IL-1β (Lafrance et al. 2010). Thus, it has been proposed that hyperleptinemia and leptin resistance may lead to a higher inflammatory response in the brain (Misiak et al. 2012).

Another protein secreted by adipose tissue is adiponectin, which is highly involved in modulation of insulin sensitivity, homeostasis of glucose and fatty acids and anti-inflammatory action (Ouchi et al. 2003). Adiponectin has also been described to have neuroprotective activities with its receptors located in the brain (Letra et al. 2014). A little literature data is available concerning the protective role of adiponectin in mild cognitive impairment. In one study reduced amounts of subcutaneous fat and low levels of plasma adiponectin were found to be associated with MCI in men (Kamogawa et al. 2010). Although the findings so far suggest that some inflammatory mediators and adipocytokines can be involved in cognitive function in elderly subjects with T2DM are limited. Therefore, the aim of the current study was firstly, to determine the serum levels of adiponectin, leptin and IL-1 β in elderly diabetic patients with and without MCI, and secondly, to examine the relationships between these three markers and clinical and cognitive parameters.

## Materials and methods

### Study population

A survey was conducted among 194 unselected elders attending an outpatient clinic belonging to the Department of Internal Medicine and Diabetology, University Hospital no 1, Lodz, Poland. A brief recruitment screening was conducted by the investigators to identify potential participants based on the following inclusion criteria: age 65 and over, diabetes type 2 diagnosed minimum 1 year earlier, ability to understand and cooperate with study procedures. The exclusion criteria were diagnosed depression or dementia, use of possible or known cognition-impairing drugs in the previous 3 month, presence of neoplasm, constant alcohol or substance abuse, severe visual, mobility, or motor coordination impairment, history of head trauma, inflammatory or infectious brain disease, severe neurological or psychiatric illness.

The first part of visit included a morning blood draw after a 10–12 h overnight fast, blood pressure measurements, height and weight assessment and complete physical examination. Capillary glucose level measurement was performed after breakfast to ensure that participants were not hypoglycemic at the time of cognitive testing. The second part of the visit took place in a private area in the clinic. The subjects completed a questionnaire describing baseline demographics and underwent cognitive testing.

### Participant characteristics, clinical evaluation and risk factor assessment

Demographic variables and possible risk factors were recorded in a standardized interview. Weight and height were measured to calculate body mass index (BMI = weight/ height^2^ [Kg/ m^2^]). A detailed medical history of diabetes type 2 was taken: diabetes duration, current treatment for diabetes and complications if present, co-morbid diseases of the patient (hyperlipidemia, hypertension, cardiovascular disease, lung disease, cancer, gastrointestinal tract diseases and other) and their treatment. Educational level was recorded in years of education. Diabetic vascular complications were assessed based on the existence of nephropathy, retinopathy, neuropathy, cardiovascular disease (CVD) and stroke. Hypertension was defined as either a history of hypertension or use of any antihypertensive agents, Hyperlipidemia defined as use of any lipid lowering agent or an untreated serum LDL cholesterol level 2.6 mmol/l or/and triglycerides 1.7 mmol/l.

### Blood biochemistry

After overnight fasting, blood samples were taken by venipuncture to assess serum levels of glycosylated hemoglobin (HbA1c), total cholesterol, triglycerides, low-density lipoprotein cholesterol (LDL-C) and high-density lipoprotein cholesterol (HDL-C). All the parameters were measured in a centralized laboratory.

### Determination of serum adiponectin, leptin and IL-1 β

The serum levels of adiponectin, leptin and IL-1 β were determined by Quantikine Human Immunoassay ELISA kit (R & D System, Minneapolis, USA) according to the manufacturer’s instructions. Minimum detectable concentrations were: 0.246 ng/ml for adiponectin, 7.8 pg/ml for leptin, and 1 pg/ml for IL-1 β.

### Neuropsychological evaluations

All participants underwent the following tests: the Montreal Cognitive Assessment (MoCA) (Nasreddine et al. 2005) to evaluate the cognitive impairment, Katz Basic Activities of Daily living (BADL) and Lawton Instrumental Activities of Daily Living (IADL) questionnaires to collect information on daily activities (Katz et al. 1970; Lawton and Brody 1969). The MoCA tests 8 cognitive domains, visual-spatial ability, attention, executive function, immediate memory, delayed memory, language, abstraction, calculation, and orientation, for a maximum total score of 30. The normal MoCA score is ≥26, with one point added if the subject has fewer than 12 years of formal education. The MoCA is better than other tools to detect MCI in the elderly patients with type 2 diabetes (Alagiakrishnan et al. 2013). MCI was diagnosed based on criteria established by the 2006 European Alzheimer’s Disease Consortium which is the currently available standard test (Petersen 2004; Portet et al. 2006). The criteria include: cognitive complaints coming from the patients or their families, the reporting of a decline in cognitive functioning relative to previous abilities during the past year by the patient or informant, cognitive disorders as evidenced by clinical evaluation (impairment in memory or in another cognitive domain, which in this study was assessed by MoCA), absence of major repercussions on daily life (measured in our study by the Katz BADL and Lawton IADL), and absence of dementia. The cut-off points for MoCA scores (19/30) are recommended for the diagnosis of ‘Dementia’- in epidemiological studies. Patients with a score of 19 or below were excluded from the study as suffering from dementia and referred to a psychiatrist for further care.

Two groups of subjects were evaluated: group 1 - patients with MCI and group 2 - patients without MCI (controls).

### Ethics

The study was operated in accordance with the World Medical Association’s Declaration of Helsinki. Each participant was identified by a number to maintain privacy. The approval was obtained from the independent local ethics committee of the Medical University of Lodz (No RNN/420/13/KB). The purpose, nature, and potential risks of the experiments were fully explained to the subjects, and all subjects gave their written, informed consent at the beginning of the study. The study included only patients who were fully able to understand and cooperate with the study procedures.

### Statistical analysis

All continues results are presented as means ± SD. Frequencies and percentages were calculated to enable comparison of characteristics between patients with MCI and controls. Normality of distributions was assessed using the Shapiro-Wilk test. The descriptive statistics for the categorical variables were tested using the χ^2^, and the continuous variables using the Student’s T-test or the Mann Whitney-U test where applicable. Relationships were assessed using the Pearson’s correlation analysis for normally distributed variables and Spearman rank correlation for non-normally distributed variables. As many factors can influence the results, the simple logistic regression was performed to select so-called independent factors which increase the selection risk of MCI in elderly patients with type 2 diabetes. The independent variables entered in the model at step one were: demographic variables (age, gender, education), duration of diabetes, glycaemic control (HbA1c level), cardiovascular diseases (MI, angina, stroke), cardiovascular risk factors (BMI, smoking status, hyperlipidaemia, previous HA or use of HA drugs), microvascular complications, presence of hypoglycemia, number of co-morbid conditions, and the levels of lipids, adiponectin, leptin and IL-1 β. We choose variables based on current literature which can potentially can influence the results (Cukierman-Yaffe et al. 2009; Umegaki et al. 2011; Misiak et al. 2012) . Multivariable regression was then used to select the “strongest” factor from the independent risk factors. All the significant variables with *p* < 0.05 included in simple logistic regression model were introduced to this analysis. The multivariable model was optimized, using a stepwise approach (backward elimination with Wald criteria). Odds ratios (OR) were computed and presented with the 95 % interval of confidence (CI). A p-value of less than 0.05 was considered statistically significant. Statistica 10.0 (StatSoft, Poland, Krakow) was used for analysis.

## Results

### The baseline characteristics of the study group

Tables 1 and 2 present the characteristics of type 2 diabetic elderly patients with and without MCI. The χ^2^ test showed that patients with MCI were more often diagnosed with CVD, hypertension, hyperlipidemia, retinopathy, nephropathy and hypoglycemia compared to controls. No significant differences were found between the groups with regard to sex, smoking habit, stroke, neuropathy, type of concomitant disease or treatment. Furthermore, the Mann–Whitney U-test and Student’s T-test showed that patients with MCI tended to be older, less educated, with a longer duration of diabetes, higher number of co-morbidities, higher BMI , higher levels of HbA1c and triglicerydes, and lower concentrations of HDL cholesterol (Table 3). MoCA score was significantly lower in subjects with MCI compared with controls. Lastly, there were no significant differences between the groups with regard to total cholesterol and LDL cholesterol levels (p > 0.05).

**Table 1:** Characteristics of type 2 diabetic elderly patients with and without MCI

Variable	Type 2 diabetes with MCI (*n* = 62)	Type 2 diabetes without MCI (*n* = 132)	χ^2^	*P* value
Gender, Female	32 (51.6 %)	50 (37.8 %)	3.26	0.07
Smoked tobacco regularly	12 (19.4 %)	34 (25.7 %)	0.96	0.32
Macrovascular complications				
Previous CVD	48 (77.4 %)	28 (21.2 %)	55.93	*P* < 0.001
Stroke	2 (3.2 %)	5 (3.78 %)	0.04	0.84
Previous HA/ use of HA drugs	60 (96.7 %)	93 (70.4 %)	17.53	*P* < 0.001
Hyperlipidemia	56 (90.3 %)	86 (65.15 %)	13.62	*P* < 0.001
Microvascular complications				
Retinopathy	43 (69.4 %)	43 (32.6 %)	23.12	*P* < 0.001
Nephropathy	32 (51.6 %)	38 (28.8 %)	9.53	0.002
Neuropathy	10 (16.1 %)	13 (9.8 %)	1.59	0.21
Treatment				
OAD	61 (98.4 %)	126 (95.4 %)	1.04	0.31
Insulin	23 (37.1 %)	47 (35.6 %)	0.04	0.84
Hypoglycemia	38 (61.29 %)	48 (36.36 %)	3.88	0.048

*MCI* mild cognitive impairment, *CVD* cardiovascular disease, *HA* hypertension, *OAD* oral anti-diabetic drug. Data are mean – SD values. χ^2^ test was used to test for significant differences

**Table 2:** Comparison of concomitant disease and drug use by type 2 diabetic elderly patients with and without MCI

Variable	Type 2 diabetes with MCI (n = 62)	Type 2 diabetes without MCI (n = 132)	χ^2^	p
Other diseases:				
Lung disease (%)	11 (17.7 %)	14 (10.6 %)	1.91	0.16
Atrial fibrillation (%)	14 (22.6 %)	22 (16.6 %)	0.98	0.32
Heart failure (%)	16 (25.8 %)	22 (16.6 %)	2.24	0.13
Gastrointerstinal tract disease (%)	29 (46.7 %)	41 (31.1 %)	4.52	0.03
Kidney disease (%)	15 (24.2 %)	24 (18.2 %)	0.95	0.33
Thyroid diasease (%)	17 (27.4 %)	30 (22.7 %)	0.51	0.48
Other treatment:				
Angiotensin-converting enzyme inhibitors (%)	35 (56.5 %)	56 (42.4 %)	3.33	0.06
Angiotensin II receptor blockers %	25 (40.3 %)	49 (37.1 %)	0.18	0.67
Diuretics (%)	23 (37.1 %)	32 (24.2 %)	15.36	*P* < 0.001
Calcium channel blockers (%)	20 (32.3 %)	39 (29.5 %)	0.15	0.7
a1-Blockers (%)	7 (11.3 %)	11 (8.3 %)	0.44	0.51
B-blockers (%)	43 (69.3 %)	48 (36.3 %)	18.44	*P* < 0.001
Antiplatelet medications (%)	48 (77.4 %)	69 (52.6 %)	10.8	0.001
Lipid-lowering medications (%)	42 (67.7 %)	89 (67.4 %)	0.01	0.96

*MCI* - mild cognitive impairment, Data are mean – SD values. χ^2^ test was used to test for significant differences

**Table 3:** Clinical characteristics and biochemical parameters of type 2 diabetic elderly patients with and without MCI

Variable	Type 2 diabetes with MCI (n = 62)	Type 2 diabetes without MCI (n = 132)	Z/t	P value
Age (years)	74.7 ± 3.9	72.5 ± 4.9	−3.39	*P* < 0.001
Education-years	9.8 ± 1.9	12.1 ± 2.2	6.72	*P* < 0.001
Duration of T2DM (years)	10.63 ± 6.2	6.45 ± 5.07	−5.59	*P* < 0.001
BMI (kg/m^2^)	29.8 ± 3.5	28.6 ± 3.1	−2.48	0.01
Adiponectin (μg/ml)	6.06 ± 4.26	10.02 ± 4.4	5.53	*P* < 0.001
Leptin (ng/ml)	25.19 ± 12.09	18.11 ± 8.26	−3.69	*P* < 0.001
IL-1 β (ng/ml)	1.59 ± 7.69	1.08 ± 5.68	−4.44	*P* < 0.001
HbA1c (%)	7.62 ± 0.69	7 ± 0.5	−5.83	*P* < 0.001
CHOL-C (mmol/L)	10.13 ± 2.2	9.58 ± 1.76	−1.86	0.06
LDL-C (mmol/L)	5.79 ± 1.49	5.64 ± 1.5	−1.15	0.24
TG (mmol/L)	10.29 ± 2.66	9.24 ± 1.79	−5.04	*P* < 0.001
HDL-C (mmol/L)	2.36 ± 0.64	2.66 ± 0.44	4.61	*P* < 0.001
Co-morbidity (n)	6.3 ± 3.06	2.8 ± 1.8	−7.26	*P* < 0.001
MoCA score	21.5 ± 1.5	27.3 ± 1.2	11.21	*P* < 0.001
Katz BADL score	4.95 ± 0.21	4.96 ± 0.19	0.11	0.9
Lawton IADL score	7.98 ± 0.12	8 ± 0.01	0.17	0.85

*T2DM* diabetes type 2, *BMI* body mass index, *CHOL* total cholesterol; *HbA1c* glycosylated hemoglobin; *HDL-C* high-density lipoprotein cholesterol; *LDL-C* low density lipoprotein cholesterol; *TG* triglycerides; *MoCA* - Montreal Cognitive Assessment; *BADL* Basic Activities of Daily living, *IADL* Instrumental Activities of Daily Living, Data are mean – SD values. Mann–Whitney U test (Z), or t test was used to test for significant differences

### Serum levels of adiponectin, leptin and IL-1 β in MCI subjects and controls

Serum levels of leptin and IL-1 β were significantly increased in patients with MCI compared to controls (*p* < 0.001). Serum concentrations of adiponectin were lower in subjects with cognitive impairment (Table 3). In the group of diabetic elderly patients with MCI adiponectin level was negatively correlated with leptin level (*r* = −0.64, *p* < 0.001), with IL-1 β level (*r* = −0.7, *p* < 0.001) and with BMI (*r* = −0.78, *p* < 0.001). Leptin concentration was positively correlated with IL-1 β level (*r* = 0.54, *p* < 0.001). Furthermore, both leptin and IL-1 β levels were highly correlated with BMI. A positive but weak correlation was found between IL-1 β level and MoCA score, and a negative correlation between adiponectin level and MoCA score. Leptin level was inversely correlated with HDL cholesterol level. The results are presented in Table 4. Since the levels of adipokines, as leptin and adiponectin, are dependent of body fat mass content and the group with T2DM and MCI had significantly higher BMI values than the other group, we divided all subjects into groups adjusted for BMI (Table 5). We observed that the adipokines results observed between the 2 groups persist significant after statistical adjustment for BMI. The only doubt occurs in group of patients with BMI below 25.9, however among subjects with MCI only 5 persons had such low BMI values therefore these data should be interpreted with caution. The majority of patients had BMI higher than 26 and in those we observed that elderly diabetic patients with MCI have higher levels of leptin and IL-1 β and lower levels of adiponectin.

**Table 4:** Relationship of serum levels of adiponectin, leptin and IL-1 β with other clinical indicators in group of diabetic elderly patients with MCI

	Adiponectin r	p	Leptin r	p	IL-1 β r	p
MoCA score	−0.3*	0.016	0.02	0.85	0.38*	0.002
HbA1c (%)	−0.18	0.14	0.12	0.33	0.07	0.56
CHOL mmol/l)	−0.06	0.63	0.02	0.84	0.001	0.98
LDL (mmol/l)	−0.11	0.09	0.09	0.44	0.12	0.34
TG (mmol/l)	−0.23	0.06	0.2	0.11	0.01	0.92
HDL (mmol/l)	0.24	0.06	−0.3*	0.017	−0.1	0.42
BMI (kg/m^2^)	−0.78*	*P* < 0.001	0.71*	*P* < 0.001	0.83*	*P* < 0.001
Adiponectin (μg/ml)	1					
Leptin (ng/ml)	−0.64*	*P* < 0.001	1			
IL-1 β (ng/ml)	−0.7*	*P* < 0.001	0.54*	*P* < 0.001	1	

*significance, *p* < 0.05; r-correlation coefficient

*BMI* body mass index; *CHOL* total cholesterol; *HbA1c* glycosylated hemoglobin; *HDL-C* high-density lipoprotein cholesterol; *LDL-C* low density lipoprotein cholesterol; *TG* triglycerides; *MoCA* Montreal Cognitive Assessment

**Table 5:** Serum levels of adiponectin, leptin and IL-1 β in type 2 diabetic elderly patients with and without MCI according to BMI values

Variable	Type 2 diabetes with MCI	Type 2 diabetes without MCI	Z/t	P value
Adiponectin (μg/ml)				
BMI ≤ 25.9	13.04 ± 3.98	13.5 ± 3.83	0.27	0.78
BMI 26–27.9*	9.37 ± 3.33	11.76 ± 3.05	2.68	0.009
BMI 28–29.9*	6.14 ± 2.85	10.38 ± 2.77	4.34	<0.001
BMI ≥ 30*	2.89 ± 1.59	5.44 ± 3.78	3.39	0.001
Leptin (ng/ml)				
BMI ≤ 25.9	7.6 ± 2.94	8.4 ± 5.28	0.32	0.74
BMI 26–27.9*	19.29 ± 5.0	15.6 ± 5.43	−2.43	0.017
BMI 28–29.9*	21.76 ± 8.65	17.94 ± 3.34	−2.11	0.04
BMI ≥ 30*	32.98 ± 11.5	27.03 ± 7.31	−2.51	0.014
IL-1 β (ng/ml)				
BMI ≤ 25.9*	8.82 ± 0.71	6.6 ± 2.23	−2.16	0.04
BMI 26–27.9	9.4 ± 0.75	9.27 ± 1.47	−0.33	0.73
BMI 28–29.9*	13.56 ± 3.44	9.96 ± 1.18	−5.17	<0.001
BMI ≥ 30*	21.97 ± 1.51	16.09 ± 8.6	−2.94	0.004

*significance, *p* < 0.05

*MCI* mild cognitive impairment, *BMI* body mass index. Data are mean – SD values. Mann–Whitney U test (Z), or t test was used to test for significant differences

### Logistic regression models

The univariate logistic regression models revealed that the variables which increased the likelihood of diagnosis of MCI in elderly patients with type 2 diabetes were: higher levels of HbA1c, leptin, IL-1 β and triglycerides, lower levels of adiponectin and HDL cholesterol, presence of CVD, hypertension, hyperlipidemia, retinopathy or nephropathy, hypoglycemia, longer duration of diabetes, increased number of co-morbidities, older age and fewer years of formal education (Table 6). Table 7 presents the risk factors for MCI according to multivariable regression. It found that the factors increasing the likelihood of having MCI in elderly patients with type 2 diabetes were fewer years of formal education, the presence of previous CVD and hypertension, increased number of co-morbidities, poorer glycaemic control (higher HbA1c level), higher levels of IL-1 β and lower level of adiponectin.

**Table 6:** Assessment results of the risk of having MCI in a simple logistic regression model in the patients with type 2 diabetes

Variables analyzed	ß	SE of ß	p value	OR	95 % CI
Age (years)*	0.1	0.03	0.003	1.1	1.03–1.18
Gender: female	0.28	0.15	0.07	1.3	0.95–1.79
Education (years)*	−0.62	0.11	*P* < 0.001	0.54	0.44–0.66
Smoked tobacco regularly	0.18	0.08	0.33	1.2	0.83–1.74
Duration of DM2 (years)*	0.13	0.03	*P* < 0.001	1.14	1.07–1.21
Previous stroke	0.4	0.08	0.84	1.08	0.47–2.5
Previous CVD*	1.27	0.18	*P* < 0.001	3.57	2.48–5.13
Previous HA or use of HA drugs*	1.26	0.37	0.001	3.54	1.71–7.35
Hyperlipidemia*	0.8	0.23	0.001	2.23	1.41–3.53
Retinopathy*	0.77	0.16	*P* < 0.001	2.16	1.56–2.99
Nephropathy*	0.48	0.15	0.002	1.62	1.18–2.22
Neuropathy	0.28	0.22	0.21	1.32	0.85–2.06
Co-morbidity (n)*	0.55	0.08	*P* < 0.001	1.74	1.48–2.04
BMI (kg/m2)	0.11	0.04	0.15	1.12	1.02–1.23
HbA1c (%)*	1.64	0.28	*P* < 0.001	5.2	2.95–9.14
CHOL mmol/l)	0.01	0.004	0.06	1.01	0.99–1.02
LDL (mmol/l)	0.01	0.006	0.51	1.004	0.99–1.02
TG (mmol/l)*	0.01	0.005	0.002	1.014	1.005–1.02
HDL (mmol/l)*	−0.07	0.01	*P* < 0.001	0.93	0.89–0.96
adiponectin (μg/ml)*	−0.21	0.04	*P* < 0.001	0.81	0.74–0.87
Leptin (ng/ml)*	0.07	0.01	*P* < 0.001	1.07	1.03–1.11
IL-1 beta (ng/ml)*	0.11	0.02	*P* < 0.001	1.12	1.06–1.17
Hypoglycemia*	1.02	0.31	0.0013	2.7	1.48–5.15

Abbreviations: *ß*: regression coefficient; *CI*: confidence interval for odds ratio; *OR*: odds ratio; *SE*: standard error; *significance, *p* < 0.05

*DM* diabetes mellitus, *CVD* cardiovascular disease, *HA* hypertension, *MCI* mild cognitive impairment, *BMI* body mass index, *HbA1c* glycosylated hemoglobin, *CHOL* total cholesterol; *HDL* high-density lipoprotein cholesterol; *LDL* low density lipoprotein cholesterol; *TG* triglycerides

**Table 7:** Assessment results of the risk of having MCI in a multivariable logistic regression model in the patients with type 2 diabetes

Variables analyzed	ß	SE of ß	p value	OR	95 % CI
Education (years)*	−0.37	0.13	0.006	0.68	0.52–0.9
Previous CVD*	0.67	0.26	0.01	1.96	1.16–3.31
Previous HA or use of HA drugs*	1.12	0.46	0.017	3.06	1.22–7.68
Co-morbidity (n)*	0.26	0.1	0.012	1.3	1.06–1.59
HbA1c (%)*	0.93	0.39	0.019	2.54	1.16–5.58
adiponectin (μg/ml)*	−0.21	0.08	0.011	0.81	0.68–0.95
IL-1 beta (ng/ml)*	0.12	0.05	0.025	1.13	1.01–1.25

Abbreviations: *ß* regression coefficient; *CI* confidence interval for odds ratio; *OR* odds ratio; *SE* standard error; *significance, *p* < 0.05

*CVD* cardiovascular disease, *HA* hypertension, *MCI* mild cognitive impairment, *HbA1c* glycosylated hemoglobin

## Discussion

Serum levels of leptin and IL-1 β were found to be significantly increased in patients with MCI compared to controls. Furthermore leptin concentration was positively correlated with IL-1 β level. These findings are consistent with the hypothesis that low-grade systemic inflammation is associated with dementia and MCI. Although the involvement of hyperleptinemia has been suggested to play a role in the pro-inflammatory state, and elevated IL-1 β levels may be associated with the deterioration of cognition in diabetes, data about these mediators in elderly MCI subjects with T2DM is poor. Our present findings go some way to addressing this paucity of evidence.

Although IL-1β is quite frequently described in Alzheimer’s disease, whereas only few reports have investigated its levels in MCI (Brosseron et al. 2014). The Sydney Memory and Aging Study found higher levels of Interleukin-1β (IL-1β), Tumor Necrosis Factor-α (TNF-α) and Interleukin 12 in the members of the non-amnestic MCI group compared to those with normal cognitive function (Trollor et al. 2010). In other studies, increased levels of TNF-α and IL-1β were found in the cerebrospinal fluid and plasma in patients with Alzheimer’s disease (Holmes et al. 2009; Forlenza et al. 2009). Inflammatory cytokines and other mediators are found in senile plaques from brains with dementia. It has been hypothesized that in a state of peripheral insulin-resistance, insulin may cross the blood brain barrier and stimulate the overproduction of proinflammatory cytokines (Misiak et al. 2012).

Another marker of low-grade inflammation is leptin. Leptin is a hormone derived from adipose tissues. Hyperleptinemia and subsequent leptin resistance are associated with T2DM and obesity (Letra et al. 2014). A body of evidence suggests that leptin, and other adipocytokines might be involved in pathogenesis of deterioration of cognition and dementia (Misiak et al. 2012; Letra et al. 2014). One study associates leptin levels with cognitive function in older adults (Holden et al. 2009). Similarly, other studies of older adults with T2DM diabetes indicate that higher leptin levels were associated with worse cognitive function in men, but were not associated with cognitive function in women (Labad et al. 2012). However, a recently published study found no association between plasma leptin level and cognitive function at baseline and over 3-year follow-up period in MCI older adults (Oania and McEvoy 2015). One study performed in middle-aged and elderly participants who were apparently healthy found no difference in leptin plasma levels between subjects with and without MCI (Kamogawa et al. 2010). Although these studies contradict our present findings, they were performed in a general, non-diabetic, population. In diabetes, leptin function may be modulated by a number of factors, including the ratio between free and bound leptin, local leptin levels, the expression of different forms of the receptors, and the presence of specific inhibitors and inflammation (Fantuzzi and Faggioni 2000). One study showed that elderly obese subjects with higher leptin levels showed concomitant greater global brain atrophy, suggesting that brain resistance to its action may develop in obese individuals despite peripheral hyperproduction of leptin (Rajagopalan et al. 2013).

Another marker of adipose tissue dysfunction is adiponectin. Adiponectin has well known protective role against insulin resistance, atherosclerosis and anti-inflammatory activity. Recent data indicates that adiponectin levels in elderly patients with T2DM seem to be closely linked to obesity and length of disease (Coimbra et al. 2014). In our study serum concentrations of adiponectin were lower in subjects with MCI. Similarly, Teixeira et al. (2013)) report lower levels of adiponectin to be associated with cognitive dysfunction, although these do not predict additional cognitive decline or conversion to dementia in this cohort of elderly subjects. They propose that decreased adiponectin may be a surrogate marker of the pathological process in Alzheimer’s Disease, linking clinical comorbidities, inflammation and cognitive dysfunction. Kamogawa et al. (2010) observed that reduced amounts of subcutaneous fat and low levels of plasma adiponectin were found to be associated with MCI in men. Adiponectin may play a protective role in the progression of early-stage cognitive impairment. It has been shown to have neuroprotective effects on hippocampal cells (Jeon et al. 2009) and a direct action on the neurons protecting human neuroblastoma cells from apoptosis (Jung et al. 2006). However, one small study found adiponectin levels to be significantly increased in individuals with MCI and AD compared to elderly controls (Une et al. 2011) and another did not reveal any significant difference in adiponectin level between AD subjects and cognitively healthy older subjects (Bigalke et al. 2011). These contradictory results may be explained by differences in sample size, characterization of the cognitive status of included subjects, presence of clinical co-morbidities, population backgrounds and study settings. Therefore, additional studies are necessary to evaluate the role of adiponectin and other adipcytokines levels in the pathophysiology of MCI and dementia.

Our present findings indicate that higher levels of IL-1 β and lower levels of adiponectin are the factors increasing the likelihood of MCI developing in elderly patients with type 2 diabetes, according to a multivariable model. Interestingly, among many other factors adiponectin and IL-1 β levels are associated with cognition independently. Increased adiponectin levels are able to decrease the expression of such inflammatory cytokines as NF-κB activation and TNF-α and may induce the production of other anti-inflammatory molecules including the IL-1 receptor antagonist and IL-10 (Misiak et al. 2012; Ouchi et al. 2003; Fasshauer et al. 2003). Therefore, lower adiponectin levels can stimulate pro-inflammatory cascades leading to the low-grade inflammation present in MCI and AD subjects. In addition, IL-1 β level was found to be inversely correlated with adiponectin level and positively associated with leptin concentration.

However, our study has some limitations that should be kept in mind when interpreting our data.

Firstly, the study population was relatively small, which may have limited our power to examine other associations.

Secondly, the study wasn’t designed as longitudinal investigation. There have been few studies in which the cognitive declines in elderly diabetics were prospectively observed. However, the precise mechanisms underlying T2DM-related cognitive dysfunction or the development of dementia have not yet been elucidated.

Thirdly, our participants were recruited from one race/ethnicity, which may limit the generalizability of findings to other populations.

Nevertheless, the results of this study suggest that further inquiry into the direction and magnitude of the pathways linking cognitive impairment and type 2 diabetes, in elderly is warranted. Further studies are required in younger people and in those without diabetes to determine the an association between low grade inflammation, adipokines and cognitive dysfunction.

In conclusion, present study suggests that those elderly diabetic patients with MCI, are more likely to have higher levels of IL-1 β and leptin and lower levels of adiponectin. Although our findings confirm the association between higher levels of low-grade systemic inflammation, adipokines and cognition, further prospective studies are needed to confirm the role of these markers in the progression to dementia.
